# New Frontiers in *Schistosoma* Genomics and Transcriptomics

**DOI:** 10.1155/2012/849132

**Published:** 2012-11-21

**Authors:** Laila A. Nahum, Marina M. Mourão, Guilherme Oliveira

**Affiliations:** ^1^Genomics and Computational Biology Group, Centro de Pesquisas René Rachou (CPqRR), Instituto Nacional de Ciência e Tecnologia em Doenças Tropicais, Fundação Oswaldo Cruz (FIOCRUZ), 30190-002 Belo Horizonte, MG, Brazil; ^2^Centro de Excelência em Bioinformática (CEBio), Fundação Oswaldo Cruz (FIOCRUZ), 30190-110 Belo Horizonte, MG, Brazil; ^3^Faculdade Infórium de Tecnologia, 30130-180 Belo Horizonte, MG, Brazil

## Abstract

Schistosomes are digenean blood flukes of aves and mammals comprising 23 species. Some species are causative agents of human schistosomiasis, the second major neglected disease affecting over 230 million people worldwide. Modern technologies including the sequencing and characterization of nucleic acids and proteins have allowed large-scale analyses of parasites and hosts, opening new frontiers in biological research with potential biomedical and biotechnological applications. Nuclear genomes of the three most socioeconomically important species (*S. haematobium*, *S. japonicum*, and *S. mansoni*) have been sequenced and are under intense investigation. Mitochondrial genomes of six *Schistosoma* species have also been completely sequenced and analysed from an evolutionary perspective. Furthermore, DNA barcoding of mitochondrial sequences is used for biodiversity assessment of schistosomes. Despite the efforts in the characterization of *Schistosoma* genomes and transcriptomes, many questions regarding the biology and evolution of this important taxon remain unanswered. This paper aims to discuss some advances in the schistosome research with emphasis on genomics and transcriptomics. It also aims to discuss the main challenges of the current research and to point out some future directions in schistosome studies.

## 1. Introduction

Schistosomatidae (Platyhelminthes: Digenea) includes several digenetic endoparasites with complex life cycles, whose developmental stages alternate between intermediate (freshwater gastropods) and definitive hosts (birds, reptiles, fishes, and mammals) [1]. These parasites differ from other blood flukes in having separate sexes. Another important feature is the increased longevity (over 5 years) of the *Schistosoma* species in the human host [1].

*Schistosoma*, the avian and mammalian blood flukes, is the best studied genus of Schistosomatidae [1]. Several species are described, six of which infect humans causing schistosomiasis: *S. haematobium*, *S. intercalatum*, *S. japonicum*, *S. malayensis*, *S. mansoni*, and *S. mekongi*. Other species are known to infect a broad range of mammals such as hippopotamus, rodents, carnivores, and ruminant animals like buffalo, cattle, goat, lechwe, and sheep. Some hybrid species are reported [1–4]. For example, *S. mattheei*, more commonly found in cattle, is believed to form hybrids between *S. mattheei* and *S. haematobium* thus increasing its snail and definitive host range [3].

Schistosomiasis, a chronic and debilitating disease, is considered by the World Health Organization as one of the most serious public health issues and the second most prevalent tropical disease with high morbidity in the world [5]. Schistosomiasis is endemic in 77 countries [6] and its transmission is dependent on the existence and distribution of intermediate hosts. It is estimated that 237 million people require treatment worldwide and that 600 to 779 million people live in endemic areas, under infection risk [6]. Yet, exacerbating this scenario, this disease is responsible for the loss of 1.7 to 4.5 millions of years of life in the world, measured by disability-adjusted life years (DALY) [7], one of the highest indexes among all the neglected tropical diseases. Control of schistosomiasis represents a great challenge and it is based on drug treatment (Praziquantel), snail control, improved sanitation, and health education [5].

The development of powerful and scalable methods to analyse nucleic acids and proteins has changed the way biological data is surveyed. The application of such technologies, together with the development of powerful computational tools and methods, have expanded our perspective of schistosome biology and allowed a better understanding of processes such as host-parasite interaction [8–10].

This paper aims to discuss some advances in schistosome research with emphasis on genomics and transcriptomics. First, we summarize the current status of sequencing projects of nuclear and mitochondrial genomes. We also discuss some aspects of evolutionary genomics and biodiversity of schistosomes. Then, we present key findings in transcriptomic analyses. Finally, we point out the main challenges of the current research and suggest some future directions in *Schistosoma* genomic and transcriptomic studies.

## 2. *Schistosoma* Genomics

In order to strengthen traditional methods and provide new information to improve success towards schistosomiasis control, the scientific community joined efforts to assess the *Schistosoma* genomic information, starting in 1994, as an initiative of WHO/TDR (UNICEF-UNDP-World Bank-WHO Special Programme for Research and Training in Tropical Diseases) [11, 12]. At that time, only a few hundred expressed sequence tags (ESTs) from *S. mansoni* were available [13]. The WHO/TDR support opened the possibility to generate data that could be translated into new tools for schistosomiasis diagnostics and treatment, representing the “kickoff” of *Schistosoma* nuclear genome studies.

### 2.1. Nuclear Genomes

The karyotype of known *Schistosoma* species comprises seven pairs of autosomes and one pair of sex chromosomes (female = ZW, male = ZZ), ranging in size from 18 to 73 megabases (Mb) [14, 15].

The nuclear genome of three *Schistosoma* species was sequenced (Table 1). The *S. japonicum* (Anhui isolate) and *S. mansoni* (Puerto Rico isolate) genomes were decoded and simultaneously published as an initiative of the Wellcome Trust Sanger Center Institute in collaboration with the Institute for Genomic Research (TIGR) and the *Schistosoma japonicum* Genome Sequencing and Functional Analysis Consortium [16, 17]. Recently, the *S. haematobium* (Egyptian isolate) genome was sequenced opening new possibilities in comparative genomics of schistosomes [18].

*S. haematobium*, *S. japonicum*, and *S. mansoni* carry a nuclear genome of 385, 397, and 363 Mb composed by approximately, 13,073, 13,469, and 10,852 protein-coding genes, respectively [16–19]. It is worth to mention that the number of predicted protein-coding genes does not reflect the genome structure per se, but the actual state of analyses of the different draft genome data.

The genetic linkage map of *S. mansoni* was obtained and allowed the refinement of the genome sequencing data providing a means for gene discovery and gene function analysis [20, 21]. More importantly, it has allowed the identification of genetic loci that determine important traits such as host specificity, virulence, and drug resistance.

In fact, although the quality and annotation of the *S. mansoni* genome sequence data have been greatly improved (see below); the corresponding data of *S. haematobium* and *S. japonicum* are still considered drafts [16–19]. Their genome sequences are distributed in a large number of contigs; therefore extensive work is required. Constant data refinement is necessary to provide a reliable comprehension of the genome and gene models and to provide a curated annotation.

The *S. mansoni* genome annotation has been improved by combining different sequencing strategies [19]. Such strategies include new assembly of existing capillary reads supplemented with an additional ~90,000 fosmid and BAC end sequences, deep sequencing of clonal DNA (NMRI strain, Puerto Rican origin) using 108-based paired reads on Illumina Genome Analyzer IIx platform, and RNA-seq data of different parasite life stages.

Progress on the knowledge of *S. mansoni* genome features is still expanding, especially with the application of the next-generation sequencing platforms and single nucleotide polymorphism typing methods. For instance, 14 new microsatellite markers were recently identified by *de novo* assembly of massive sequencing reads; these new features can be employed for pedigree studies [22]. Yet, newer sequencing technologies will allow for the development of population genomics studies of field, laboratory, and clinical isolates.

As a result of the availability of the *Schistosoma* genome sequence data, comparative analyses across different species became possible bringing together different areas of research [21, 23, 24]. Comparative genomics of three *Schistosoma* species revealed several conserved features such as the overall GC content, sequence identity, presence of repetitive elements, and synteny [18]. This genome-wide analysis corroborates previous genetic studies and supports the evolutionary hypothesis of *S. haematobium* and *S. mansoni* as the most related species, followed by *S. japonicum*, to the exclusion of other Trematoda such as *Clonorchis*, *Fasciola*, and *Schmidtea*.

### 2.2. Mitochondrial Genomes

Mitochondrial genes have been used as molecular markers for species and strain identification, which is key to a variety of studies such as phylogenetics, population genetics, biogeography, and molecular ecology [25–29]. Besides helping to elucidate the evolutionary relationships among taxa, mitochondrial genes have been successfully applied into epidemiological studies, monitoring, and control of microbes, parasites, and vectors of medical and socioeconomic importance. The analysis of the whole mitochondrial genome of diverse taxa has changed the perspective of such studies.

The mitochondrial genome of six *Schistosoma* species was sequenced (Table 1) including *S. haematobium* (NC_008074), *S. japonicum* (NC_002544), *S. mansoni* (NC_002545), *S. malayensis*, *S. mekongi* (NC_002529), and *S. spindale* (NC_008067) [27, 30–32]. These genomes range in size from 13,503 to 16,901 bp and encode 36 genes comprising two ribosomal genes (large and small subunit rRNA genes), and 22 transfer RNA (tRNA) genes, as well as 12 protein-encoding genes (atp6, cob, cox1–3, nad1–6, and nad4L). The atp8 gene, commonly found in other phyla, is absent from Platyhelminthes, whose mitochondrial genomes have been analysed [31]. Moreover, each *Schistosoma* mitochondrial genome contains a long noncoding region that is divided into two parts by one or more tRNA genes, which is found in other Platyhelminthes and vary in length according to each taxon.

Comparative mitogenomics have revealed a highly conserved gene order among distinct Platyhelminthes with the exception of some *Schistosoma* species [25, 30–33]. The mitochondrial genome of the Asian schistosomes, *S. japonicum* and *S. mekongi*, displays the same gene order as other Digenea and Cestoda [25, 32].

In contrast, the gene order in the mitochondrial genome of *S. haematobium*, *S. mansoni*, and *S. spindale* is strikingly different from other taxa [25, 30]. The unique gene order rearrangements identified in these species constitute valuable information to the reconstruction of the phylogenetic relationships of *Schistosoma* and other Platyhelminthes (Section 3.1).

## 3. Evolutionary Genomics and Biodiversity

### 3.1. Evolutionary Genomics of *Schistosoma*

The use of genomic data is valuable to the understanding of the evolutionary history of *Schistosoma* especially due to the absence of fossil records. Molecular markers have been used to test hypotheses in a variety of *Schistosoma* phylogenetic, phylogeographic and epidemiological studies [4, 25, 34–36]. Traditionally, these markers include the nuclear ribosomal RNA genes (18S, 5.8S, and 28S) and the internally transcribed spacer (ITS) region besides a number of mitochondrial genes (cob, cox1–3, nad1–6, etc.).

Based on mitochondrial data from different studies, a standard phylogeny of 23 *Schistosoma* species was proposed [25]. Six clades were identified that correlate with the geographic distribution of the species analysed. These clades include (1) the *S. japonicum* complex (*S. japonicum*, *S. malayensis*, *S. mekongi*, *S. ovuncatum*, and *S. sinensium*) found in Central and South Eastern Asia; (2) the *S. hippopotami* clade (*S. edwardiense* and *S. hippopotami*) distributed in Africa; (3) the proto-*S. mansoni* clade (*S. incognitum* and *S. turkestanicum*) in Central Asia, Near East, and Eastern Europe; (4) the *S. mansoni* clade (*S. mansoni* and *S. rodhaini*) found in Africa and South America; (5) the *S. haematobium* clade (*S. bovis*, *S. curassoni*, *S. guineensis*, *S. haematobium*, *S. intercalatum*, *S. kisumuensis*, *S. leiperi*, *S. margrebowiei*, and *S. mattheei*) occurring in different parts of Africa; (6) the *S. indicum* clade (*S. indicum*, *S. nasale*, and *S. spindale*) found in India and South Asia.

There are divergent opinions concerning the origin of the genus *Schistosoma* and its intermediate snail host [25, 37–39]. The “out of Asia” hypothesis is the most generally accepted, indicating a migration followed by dispersion of these parasites from Asia to Africa [25]. In this regard, studies of molecular phylogeny suggested that the genus *Schistosoma* had its origin in Asia and (at least two descendants) colonized the African continent independently, where they easily radiated, becoming exclusive parasites of planorbid snails. Returning to Asia, the parasites diversified into groups of species, characterized by the position of the spike in the eggs [38]. The molecular phylogeny of seven *Schistosoma* species suggested that schistosomes were endoparasites of rodents and ruminants and were transmitted to the first hominids in Africa as a migration to savanna areas occurred [40]. However, in order to better define the *Schistosoma* origin, there is a need to sample a broader range of species.

Most of the widely accepted *Schistosoma* phylogenies are primarily based on sequence alignments of mitochondrial genes such as cox1–3, and nad1–6, [25, 30]. Other studies use changes in the mitochondrial gene order as phylogenetic markers. Indeed, these gene rearrangements, considered to be rare evolutionary events, have been applied to an increasing number of studies (cf. [41]).

As mentioned before, major changes in the mitochondrial gene order were identified in some *Schistosoma* species (Section 2.2), more specifically in the African clades (*S. mansoni* and *S. haematobium*) and the *S. indicum* group (*S. spindale*). This provides further evidence for the Asian ancestry of schistosomes and corroborates the concept of the reinvasion of Asia by members of the *S. indicum* group from Africa [27, 30].

Besides reconstructing the phylogenetic relationships of diverse taxa, the evolutionary framework has been applied to improve functional annotation of genes and gene products, as well as to study gene/protein family evolution [41]. Phylogenomics has allowed the identification and characterization of all eukaryotic protein kinases encoded in the *S. mansoni* genome and improved the functional annotation of over 40% of them, highlighting the molecular diversity of these enzymes [42, 43].

A phylogenetic analysis was also employed in the classification of *S. mansoni* histone proteins, which play a key role in epigenetic modifications that might reflect the parasite complex lifestyle [44]. By applying an evolutionary framework to analyse a large sequence dataset from a broad range of organisms, functional annotation of the *S. mansoni* histone proteins was improved.

Moreover, phylogenomics has unraveled the distinct evolutionary histories of three expanded endopeptidase families in *S. mansoni* [45]. This analysis included members of the metallopeptidase M8, serine peptidase S1, and aspartic peptidase A1 families, which were originated from successive events of gene duplication followed by divergence in the parasite lineage after its diversification from other metazoans.

Other protein families such as nuclear receptors and antioxidants have been characterized in *S. mansoni* by applying evolutionary and functional approaches [46, 47]. The identification of SmNR4A, a member of the nuclear receptor subfamily 4, and its relatedness to the human homologs were corroborated by phylogenetic analysis [46]. This study also demonstrated that SmNR4A expression was regulated throughout parasite development.

Thioredoxin and glutathione systems, involved in a variety of cellular processes including antioxidant defense, differ in parasitic and free-living Platyhelminthes [47]. By applying different phylogenetic methods and experimental testing, it has been shown that the canonical enzymes of such systems were lost in the parasitic lineages.

### 3.2. DNA Barcoding for Biodiversity Assessment

The availability of sequencing data from mitochondrial genomes from closely related taxa to *Schistosoma* has provided a basis for studies ranging from phylogenetic systematics to molecular ecology and so forth [27, 29, 48, 49]. Importantly, it allowed the assessment of molecular markers prior to broad scale sampling by comparing data from individual genes versus completely sequenced mitochondrial genomes.

Data from African and Asian schistosomes were compared to an *S. mansoni* intraspecific dataset showing a positive correlation between polymorphism and species divergence [27]. A positive correlation rather than random distribution was identified for all mitochondrial genes with the exception of cox1 and nad1. Furthermore, partial sequences of cox1 have been shown not to be the ideal marker for either species identification or population studies of *Schistosoma* species. Instead, authors have suggested the use of cox3 and nad5 sequences for both phylogenetic and population studies of such species [27].

DNA barcoding was performed for samples of *S. haematobium* using cox1 and nad1 as molecular markers [49]. The results supported the identification of group 1 and 2 haplotypes based on phylogenetic analysis. Moreover, no change in genetic diversity was detected across samples collected over different time points. This approach allowed the development of new assays based on group-specific PCR primers and SNaPshot probes to assist genetic screening of schistosome isolates.

Another study employing mitochondrial sequences was performed in *Schistosoma* cercariae harvested from *Biomphalaria choanomphala* as part of parasitological and malacological surveys for *S. mansoni* across Lake Victoria [48]. DNA barcoding of cercarial samples revealed the presence of hybrid species between *S. mansoni* and *S. rodhaini*, which is typically found in small mammals. Moreover, the phylogenetic analysis identified a new sublineage within *S. rodhaini*.

DNA barcoding studies are underway for an increasing number of *Schistosoma* species (Table 1), whose data is made available through the Barcode of Life Database (BOLD) system (http://www.boldsystems.org/). This initiative includes contributors from the Biodiversity Institute of Ontario, Canada, and the University of New Mexico, USA, among others. Other Schistosomatidae genera are subject to DNA barcoding analysis such as *Allobilharzia*, *Austrobilharzia*, *Bilharziella*, *Bivitellobilharzia*, *Dendritobilharzia*, *Gigantobilharzia*, *Griphobilharzia*, *Heterobilharzia*, *Macrobilharzia*, *Orientobilharzia*, *Ornithobilharzia*, *Schistosomatium*, and *Trichobilharzia*.

Advances in DNA barcoding of a broader range of Schistosomatidae will create a framework for species identification from environmental and clinical samples as well as specimens deposited in biological collections. Moreover, DNA barcoding analysis will help reconstructing the evolutionary relationships across different Platyhelminthes and ultimately improve our understanding of parasite biology and evolution.

## 4. *Schistosoma* Transcriptomics

Transcriptomic analyses are valuable not only for studies of differential regulation in gene expression, but also for the identification of splicing variants. These approaches have proved to be essential in the identification of gene-coding regions during the process of genome annotation.

In this section, we aim to review the achievements in *Schistosoma* transcriptomic studies that range from the whole organism and tissue analyses to those performed at the cellular level. Transcriptomics in physiological conditions and in response to a variety of treatments are investigated with respect to the interaction with the host as well as other environments. This section summarizes the accomplishments of gene function studies and the diverse techniques that are being employed.

### 4.1. *Schistosoma* Transcriptomics

A fine tuning in gene expression of parasites must occur to enable them to survive in such a complex and extremely diverse milieu, adapting to diverse environments as evading the immune system and, at the same time, exploiting different hosts. Transcriptomic studies came along allowing a temporal and quantitative analyses of gene expression, which are crucial to completely exploit different datasets and assess parasite biology at the molecular level.

Aiming to elucidate the differential regulation on the *Schistosoma* transcriptomics, a large number of projects employed diverse strategies, such as the ones using expressed sequence tags (ESTs) [50], open reading frame expressed sequence tags (ORESTEs) [51], and serial analysis of gene expression (SAGE) [52, 53].

The increasing number of projects resulted in the production of a vast amount of sequence data and their availability in public databases. It is noteworthy that only *S. japonicum*, *S. mansoni* and, not until very recently, *S. haematobium* had some of their transcriptome explored (Table 1).

### 4.2. Exploring the Transcriptome

The large number of transcriptome projects accelerated the studies in this area by providing means to the use of complementary approaches, especially microarray analysis. Together, these approaches have been used to answer a wide range of questions. In the early years, microarray datasets conceived the profile of gene expression between genders of *Schistosoma* species such as *S. japonicum* and *S. mansoni* [54–56]. This greatly extended the number of known sex-associated genes and also provided new insights into changes in gene expression promoted by parasite pairing. In addition, gender biased alternative splicing patterns were observed, which are perhaps involved in the generation and maintenance of the sexual dimorphism [57].

Later, studies profiled the *S. mansoni* gene expression in different life stages broadening our understanding of factors controlling schistosome development [8, 53, 58]. While comparing different parasite life stages, information acquired from microarray datasets highlighted putative antischistosome candidate molecules to be used as vaccine targets including Dyp-type peroxidases, fucosyltransferases, G-protein coupled receptors, leishmanolysins, tetraspanins, and the netrin/netrin receptor complex [59].

Some of these candidates (fucosyltransferases, leishmanolysins, and tetraspanins) are members of protein families, which are expanded in the *S. mansoni* predicted proteome in comparison with other metazoans as inferred by phylogenomic analysis (http://phylomedb.org/). Leishmanolysins, which are members of the metallopeptidase M8 family, were analysed in detail corroborating the potential of these enzymes as future therapeutic targets [45].

Another study identified upregulated genes during the first five days of schistosomula development *in vitro* that are involved in blood feeding, tegument and cytoskeletal development, cell adhesion, and stress responses [8]. The highly upregulated genes included a tegument tetraspanin Sm-tsp-3, a protein kinase, a novel serine protease and serine protease inhibitor, and intestinal proteases belonging to distinct mechanistic classes. Other groups have analysed immunity towards *Schistosoma* using protein microarrays as a vaccine discovery tool. They selected a subset of proteins from genomic, transcriptomic, and proteomic data to perform experimental validation aiming at identifying novel antischistosome vaccine candidates [60, 61].

Recently, an elegant approach to investigate specific tissues such as gastrodermis and both genders reproductive tissues was performed by applying laser microdissection microscopy combined to microarray. These studies brought the perspective of expediting tissue-specific profiling [62, 63].

Additionally, expression profiles of *S. japonicum* were explored in studies comparing two Chinese isolates (Anhui and Zhejiang), as well as the Anhui Chinese and Philippines isolates [55, 64]. Later, an interspecies study compared the *S. japonicum* and *S. mansoni* transcriptomes. Surprisingly, despite their large phenotypic differences, there was a reduced number of differentially expressed genes based on the expression profile, which might reflect a limitation of the technique (preferential hybridization related to species polymorphisms) [8]. Bypassing these pitfalls, the next generation sequencing platforms allow exploiting expression data (RNA-seq) of any organism without previous knowledge of the genome or transcriptome and, further, will reveal new features of the parasite transcriptional landscape. 

The most recent achievement in this area was the refinement of the genomics and transcriptomics data using a combination of Sanger capillary sequencing, next generation DNA sequencing, genetic markers, and RNA-seq data from several *S. mansoni* life stages [19]. A previous study, explored the transcriptome of *S. mansoni* adult males obtaining 11 novel microexon genes (MEGs), as well as mapping 989 and 1196 contigs to intergenic and intronic genomic regions, respectively. This data could represent new protein-coding genes or noncoding RNAs (ncRNAs) [65].

Together, these approaches have significantly advanced our understanding of the dynamics parasite transcriptomes [9, 65]. However, there is a need to polish and translate sequence data and gene expression profiles into functional information by computational predictions and experimental characterization. The vast amount of available data opened a myriad of possibilities towards the elucidation of gene functions. It is time for data integration.

### 4.3. Posttranscriptional Mechanisms

Transcriptomics is also a key approach to study posttranscriptional regulatory mechanisms such as alternative and transsplicing [57, 66–68]. It is known that schistosomes use such mechanisms to keep their intricate life cycle.

Alternative splicing of transcripts of secreted proteins, like MEGs and polymorphic mucin isoforms, has been largely studied in schistosomes [51, 66]. This posttranscriptional processing has been proposed as a distinct genetic system to generate protein variation that would allow parasite adaptation to the immune response of different hosts. Additionally, alternative splicing has been shown to generate protein variation involved in transcriptional control and splicing [57]. In the case of SmCA150, a spliceosome interacting transcriptional cofactor, it could impact many other transcripts leading to several effects in the parasite, especially in sexual dimorphism.

Spliced leader (SL) transsplicing has been shown to be an important posttranscriptional regulation mechanism for *S. mansoni* [69]. At least 11% of all transcripts has been described as undergoing transsplicing in this organism [19]. SL-RNAs are small ncRNAs, products of small intronless genes, transcribed by DNA polymerase II and tandemly repeated in the genome [67].

Differently from nematodes, *S. mansoni* carries a single SL sequence, which is composed by 36 nucleotides with an 3′-terminal AUG codon. Although the role of transsplicing is poorly understood, there is an evidence suggesting that SL transsplicing would function providing an initiator methionine for translation initiation of some recipient mRNAs in Platyhelminthes [68]. This mechanism is also involved in the resolution of polycistronic operons in monocistronic transcripts in *S. mansoni* [19, 70].

### 4.4. Transcriptome Data and Gene Function

Despite the great potential revealed by the increasing number of genome and transcriptome studies in schistosomes, there are still several *Schistosoma* species that remain neglected. In contrast to the vast amount of data available in public databases, limited data has been decoded in functional studies and/or in the development of successful tools to fight schistosomes. This slow evolving scenery might be due to several reasons. Some of them include the convolution of schistosomes life cycle, the complex interaction with their hosts, time and labor-demand functional studies, the difficulties to nurture the complete life cycle *in vitro*, and the absence of immortalized cell lines, which has delayed the development of transgenesis tools and models [71].

Lately, the posttranscriptional suppression of genes through RNA interference (RNAi) techniques has been a promise to fetch progress on new interventions for schistosomiasis and other helminthiases. In 2003, two pioneer investigations marked the use of this technique in *S. mansoni* research. Simultaneously gene knockdown of glyceraldehyde-3-phosphate dehydrogenase (GAPDH) and a glucose transporter (SGTP1) in the larval stage (sporocysts) [72] and cathepsin B in adult worms [73] of *S. mansoni* were reported.

Currently, this powerful technique is widely used in schistosomes and has been employed in two studies of functional screening. The first study encompassed the exposure of sporocysts by soaking parasites to dsRNA targeting 33 genes, from calcium binding proteins, transcription factors, to receptors, and antioxidant enzymes [74]. In this work, despite the efficacy of this tool to deliver suppression of 11 different genes, it showed that, in *S. mansoni*, there is a gene-to-gene variability and exposure to dsRNA could trigger gene specific upregulation. Later, in a different study using electroporation to expose schistosomula to dsRNA of 11 target genes (expressed in distinct tissues), the efficiency of RNAi in a target specific fashion was observed [75]. Other issues with the use of RNAi have been widely discussed, not only in schistosomes, but also in other helminth parasites, showing that care should be taken when conducting and evaluating such experiments [76].

Despite the difficulties in exploring gene function by RNAi in schistosomes, many interesting advances have been achieved. Many proteases (cathepsins and aminopeptidases) [77–79], kinases [80], multidrug resistance transporters [81], and type V collagen [82], among other proteins have been studied in diverse life stages of *S. japonicum* and *S. mansoni*.

Recently, this tool has been described targeting tetraspanin 2 in diverse developmental stages of *S. haematobium* indicating the RNAi feasibility in this species [83]. Tetraspanins belong to a family of integral membrane proteins present in the outer surface membranes of the parasite tegument. Studies have shown that tetraspanins play important roles in the tegument development, maturation, or stability [84].

Importantly, the knockdown of peroxiredoxin [85] and thioredoxin glutathione reductase [86], antioxidant enzymes, were amongst the first reports of lethality after gene silencing in the parasite, demonstrating the potential of RNAi on drug target discovery. Moreover, enzymes such as glutathione-S-transferases (GST26 and GST28), peroxiredoxins (Prx1 and Prx2), and superoxide dismutase have been shown to play important roles in the protection of *S. mansoni* sporocysts in the host-parasite interplay. Knockdown of those targets increased parasite susceptibility to oxidative stress and to intermediate host immune cells as described elsewhere [87].

Recently, significant progress has been made in the area of schistosome transgenesis. Researchers are developing new tools for the introduction and expression of homologous or heterologous gene constructs via lentiviruses, replication-defective retroviruses, transposons, retrotransposons, and DNA plasmid vectors [9]. Sophisticated systems applied to assess gene function in the parasite are being achieved [88–91]. To date, eggs and miracidia are indicated as the preferred life stages for successful transformation approaches in order to enter the germline [92].

In summary, results from such independent studies have shown that schistosome transgenesis works in all the developmental stages tested and that some success has been achieved in integrating genes into the germline [88–92]. Thus, genetic knockdown experiments in schistosomes are possible.

## 5. Genomic and Transcriptomic Databases

Altogether, the increasing information on schistosome genomes and transcriptomes is now raising the possibilities to better explore molecular techniques in the field of parasitology, genomics, and computational biology allowing the development of approaches to unravel mechanisms of parasite infection, host-parasite interaction, and so forth [21, 23, 24].

In order to accelerate both parasite research and development of effective tools for schistosomiasis diagnostics and treatment, user-friendly databases have been created and made publicly available to the scientific community [9, 93–95]. Here, we highlight some of these major resources.

SchistoDB integrates genome and proteome sequence data along with functional annotation of genes and gene products of *S. haematobium*, *S. japonicum*, and *S. mansoni* [93]. This resource also covers results from large-scale analysis including ESTs, metabolic pathways, and candidate drug targets.

HelmCoP (Helminth Control and Prevention) integrates functional, structural, and comparative genomics data of helminths from different taxonomic groups and distinct hosts (plant, animal, and human) [94]. HelmCoP offers a comprehensive suite of structural and functional annotations to support comparative analyses and host-parasite interaction studies.

GeneDB is an annotation database that brings together data from a broad range of pathogens and closely related organisms [95]. GeneDB offers database-driven annotation tools and pipelines and includes manually curated information maintained by computational and biological scientists.

Collectively, these computational resources support biological and biomedical research and open new frontiers in evolutionary and functional genomics, transcriptomics, and proteomics of *Schistosoma* species and other parasites. Besides biodiversity assessment at the molecular level, these resources provide a framework for the identification and prioritization of vaccine and drug targets against human diseases. As the scientific community progresses in data integration and interpretation, we will meet the goals of current and future initiatives to improve human health [9, 12, 23, 24].

## 6. Conclusions and Future Directions

The -omics studies came along with the aim to fill the gaps of long-established approaches, enabling the use of a broad range of experimental and computational methods and contributing to dissect the biological basis of host-parasite interaction, diagnostics improvement, and the discovery of new drug and vaccine targets.

Nuclear and mitochondrial genomes of some *Schistosoma* species were completely sequenced opening new frontiers for comparative analyses, which will improve our understanding of parasite biology and evolution. Other genomes to be analysed include *Schistosoma* species from each of the six clades currently recognized, and other representatives of the Schistosomatidae. Improving accuracy/quality of genome assembly and annotation will be crucial for such analyses.

Despite the efforts in the characterization of genomes and transcriptomes of distinct *Schistosoma* species, many questions regarding the biology and evolution of this important taxon remain unanswered. These difficulties might be due to the size of schistosome genomes (~10-times larger compared to protozoan parasites), the complexity of its life cycle, the presence of rare features in posttranscriptional regulation (e.g., transsplicing), and little integration of the current information available, among other reasons.

There is an urgent need for data integration to help interpreting the massive amount of data generated over the years in this and other areas or research. The need for biocuration is essential to guide this process. We envision that, in the future, more transcriptomic and proteomic data will be used to improve the understanding of the origin and evolution of Schistosomatidae. The analysis of structural data generated by experimental or computational approaches will also shed light in the biodiversity of schistosomes at the molecular level.

In conclusion, the availability of schistosome genome and transcriptome data has provided an unprecedented resource for many research areas. Limitations do exist and should be addressed to allow advances in understanding schistosome biology and evolution and ultimately support vaccine and drug development against schistosomiasis.

## Figures and Tables

**Table 1:** Availability of genomic and transcriptomic data for *Schistosoma* species.

Taxid	Taxon	ncDNA	mtDNA	ESTs	Barcoding
6184	*Schistosoma bovis *	No	No	No	Yes
6186	*Schistosoma curassoni *	No	No	No	Yes
230327	*Schistosoma edwardiense *	No	No	No	Yes
393876	*Schistosoma guineensis *	No	No	No	Yes
6185	*Schistosoma haematobium *	Yes	Yes	Yes	Yes
157462	*Schistosoma hippopotami *	No	No	No	Yes
198245	*Schistosoma incognitum *	No	No	No	Yes
216970	*Schistosoma indicum *	No	No	No	Yes
6187	*Schistosoma intercalatum *	No	No	No	Yes
6182	*Schistosoma japonicum *	Yes	Yes	Yes	Yes
646316	*Schistosoma kisumuensis* (*)	No	No	No	Yes
216972	*Schistosoma leiperi *	No	No	No	Yes
53353	*Schistosoma malayensis *	No	Yes	No	Yes
6183	*Schistosoma mansoni *	Yes	Yes	Yes	Yes
48269	*Schistosoma margrebowiei *	No	No	No	Yes
31246	*Schistosoma mattheei *	No	No	No	Yes
38744	*Schistosoma mekongi *	No	Yes	No	Yes
216971	*Schistosoma nasale *	No	No	No	Yes
6188	*Schistosoma rodhaini *	No	No	No	Yes
191505	*Schistosoma sinensium *	No	No	No	Yes
230328	*Schistosoma* sp. Uganda-JATM-2003	No	No	No	Yes
6189	*Schistosoma spindale *	No	Yes	No	Yes

Taxid: NCBI taxonomy identifier. ncDNA: nuclear DNA genome (SchistoDB.org). mtDNA: mitochondrial DNA genome (NCBI Organelle Genome Resources). ESTs: data from NCBI dbEST (release 120701, July 1, 2012). Barcoding: DNA barcoding used on cox1 sequences (BOLD Systems). *Schistosoma sp. BH-2009.

## List of Abbreviations

atp6: ATP synthase F0 subunit6
atp8: ATP synthase F0 subunit 8
bp: Base pairs
cob: Apocytochrome b
cox1: Cytochrome c oxidase subunit 1
cox1–3: Cytochrome c oxidase subunit 1 to 3
DALY: Disability-adjusted life years
dbEST: Database of expressed sequence tags
dsRNA: Double strand DNA
ESTs: Expressed sequence tags
MEGs: Microexon genes
nad1–6: NADH dehydrogenase subunit 1 to 6
nad4L: NADH dehydrogenase subunit 4L
ncRNAs: Noncoding RNAs
ORESTEs: Open reading frame expressed sequence tags
RNAi: RNA interference
RNA-seq: RNA sequencing
SAGE: Serial analysis of gene expression
SL: Spliced leader
TDR: Special Programme for Research and Training in Tropical Diseases
UNDP: United Nations Development Programme
UNICEF: United Nations Children's Fund
WHO: World Health Organization
